# Mitotic Spindle Disruption by Alternating Electric Fields Leads to Improper Chromosome Segregation and Mitotic Catastrophe in Cancer Cells

**DOI:** 10.1038/srep18046

**Published:** 2015-12-11

**Authors:** Moshe Giladi, Rosa S Schneiderman, Tali Voloshin, Yaara Porat, Mijal Munster, Roni Blat, Shay Sherbo, Zeev Bomzon, Noa Urman, Aviran Itzhaki, Shay Cahal, Anna Shteingauz, Aafia Chaudhry, Eilon D Kirson, Uri Weinberg, Yoram Palti

**Affiliations:** 1 Novocure Ltd. Topaz Building, MATAM center Haifa 31905, Israel

## Abstract

Tumor Treating Fields (TTFields) are low intensity, intermediate frequency, alternating electric fields. TTFields are a unique anti-mitotic treatment modality delivered in a continuous, noninvasive manner to the region of a tumor. It was previously postulated that by exerting directional forces on highly polar intracellular elements during mitosis, TTFields could disrupt the normal assembly of spindle microtubules. However there is limited evidence directly linking TTFields to an effect on microtubules. Here we report that TTFields decrease the ratio between polymerized and total tubulin, and prevent proper mitotic spindle assembly. The aberrant mitotic events induced by TTFields lead to abnormal chromosome segregation, cellular multinucleation, and caspase dependent apoptosis of daughter cells. The effect of TTFields on cell viability and clonogenic survival substantially depends upon the cell division rate. We show that by extending the duration of exposure to TTFields, slowly dividing cells can be affected to a similar extent as rapidly dividing cells.

The biologic effects of electric field application on cells and living tissue have been well described in the literature^1^,^2^. Alternating electric fields have been shown to induce a wide range of frequency dependent effects on living cells. At low frequencies (under 1 kHz) alternating electric fields stimulate nerves and muscles by depolarizing the cell membrane. In addition, low frequency or pulsed electric fields have been shown to accelerate fracture healing^3^,^4^. Exposure of cells to high intensity (kV/cm) and high frequency fields in the MHz or GHz range causes heating, membrane disruption, electroporation and cell death^2^. Electric fields of intermediate frequency (10 kHz to 1 MHz) were long considered to have no significant influence on biological processes as their alternation is too rapid to cause nerve-muscle stimulation and at low intensities cause minimal heating^5^. It is only in recent years that the biological effects of intermediate frequency fields have been described. Electric fields in the frequency range of 100–500 kHz were found to have a profound inhibitory effect on the growth rate of a variety of cancer cell lines both *in vitro* and *in vivo*^6^,^7^,^8^. This has subsequently led to the development of Tumor Treating Fields (TTFields) therapy. TTFields are low-intensity (1–3 V/cm) intermediate-frequency (100–300 kHz), alternating electric fields. Clinical trials have demonstrated the effectiveness and safety of continuous TTFields treatment in patients with glioblastoma and in patients with non-small cell lung cancer^9^,^10^,^11^.

Several hypotheses to explain the mechanistic basis for the anti-cancer effects of TTFields have been proposed. A commonality among these hypotheses is the assumption that electric fields exert directional forces on polar intracellular elements such as organelles and macromolecules. A conspicuous structural feature of mitotic cells is the presence of highly polar and dynamic spindle microtubules^12^,^13^, thus potentially rendering them susceptible to the effects of an externally applied electric field. According to one hypothesis, TTFields interfere with the proper formation of the mitotic spindle, eventually activating the spindle assembly checkpoint (SAC) and consequently triggering apoptosis in a manner similar to that observed in studies with classical anti-microtubule agents (i.e. vinca alkaloids, taxanes, and epothilones)^14^,^15^,^16^,^17^. An alternative hypothesis purports that non-uniform fields are induced within mitotic cells due to the hourglass cellular structure they assume following anaphase. These non uniform fields can induce dielectrophoretic forces pushing charges and dipoles toward the cleavage furrow^6^. These hypotheses are based on the observation that cells exposed to TTFields enter mitosis but the duration of the mitotic phase is prolonged, and may eventually lead to cell death^7^. In addition, abnormal membrane blebbing, which is typically associated with apoptotic cell death^18^, is evident following these disrupted mitotic events^7^.

Another key consideration is the downstream physiological effects of putative mitotic spindle assembly disruption. The mitotic spindle governs the requisite capturing, alignment, and segregation of chromosomes to two daughter cells^19^,^20^. Missegregation of chromosomes results in aneuploidy, which can lead to genomic instability and subsequent cell death or senescence in a process termed mitotic catastrophe^21^. The SAC, also known as the mitotic checkpoint, is responsible for delaying the irreversible transition from metaphase to anaphase in order to avoid erroneous segregation of sister chromatids^22^,^23^,^24^. Anti-microtubule agents are thought to delay mitotic progression by activating the SAC, thereby inducing mitotic arrest and cell death directly from mitosis^24^. In addition, cell death can also occur following cell division^21^. For example, cycle arrest can be followed by senescence or apoptosis occurring in the subsequent G1 phase^25^,^26^,^27^,^28^. Alternatively mitotically arrested cells can escape mitosis to enter G1 phase as polyploid cells, in a process known as mitotic slippage^26^,^29^,^30^. Senescence, cell death via apoptosis, and continued cell cycling are all possible outcomes of mitotic slippage^29^.

The primary objective of this study was to determine, firstly whether and to what extent, TTFields interfere with normal formation of the mitotic spindle and secondly, to evaluate the possible contribution of impaired microtubule dynamics to this effect. We also investigated whether such disruption of the mitotic spindle can lead to subsequent chromosome missegregation and trigger cell death to avoid genomic instability. Our previous observations suggest a cell type–specific optimal effective frequency for TTFields therapy^6^,^31^. The frequencies that were chosen for this study are derived from frequency titration experiments which evaluated treatment efficacy based on cell counts. The frequencies that led to the highest reduction in cell counts are summarized in Table 1. Field intensity of 175 V/m was used for all studies, and reflects the average intensities in the clinical settings as simulated in a brain model^6^,^32^,lung model^31^,^33^, pancreas, and ovarian models (unpublished data).

Here we demonstrate that TTFields reduce the fraction of polymerized microtubules and disrupt the mitotic spindle structure, thereby leading to both cell death and to chromosome missegregation. We show that aneuploidy and multi nucleation are commonly observed in TTFields-exposed cells and thus, can plausibly explain the long term effects of TTFields exposure on clonogenic survival. Our findings suggest that since the effect of TTFields on viability and clonogenic survival substantially depends on cell division rate, extending the duration of exposure can enhance treatment efficacy in slow dividing cells.

## Results

### TTFields Interfere With Mitotic Spindle Structure

**Figure 1:**
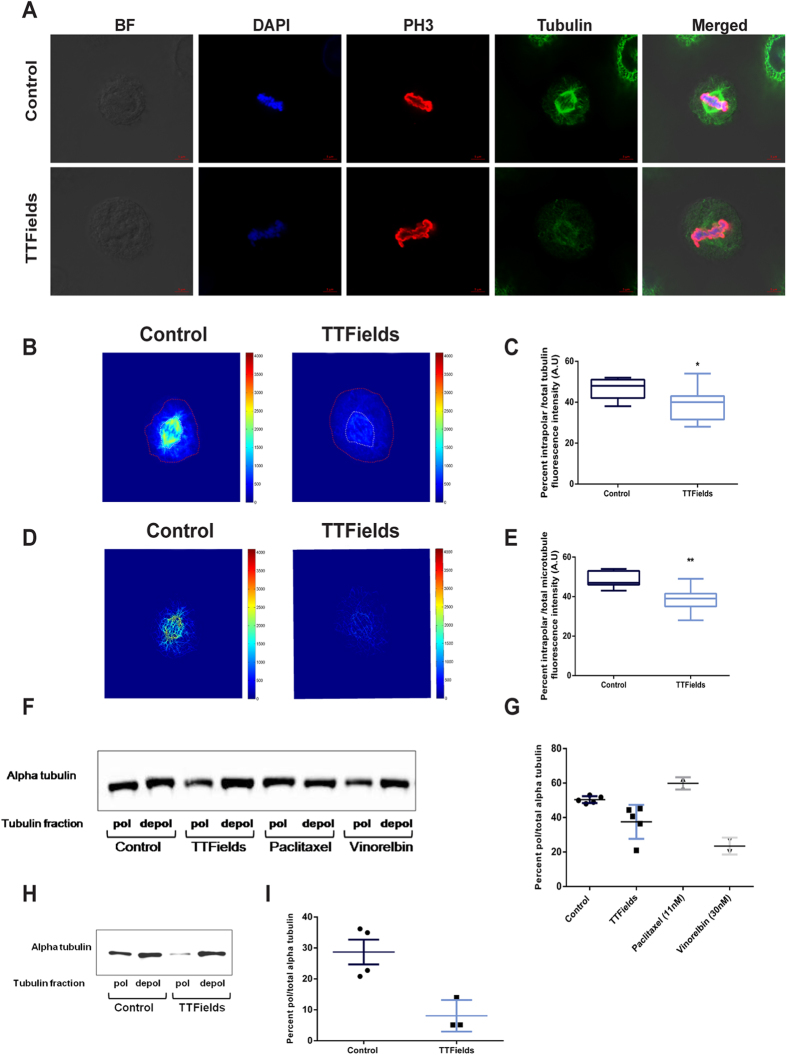
TTFields Treatment Induce Severe Mitotic Spindle Damage in Cancer Cell Lines. (A–C) A549 cells were treated with TTFields for 24 hours. (**A**) Confocal fluorescence microscopy images of: Upper panel- normal metaphase alignment of chromosomes in control cell, Lower panel: metaphase alignment in TTFields treated cell. Blue, Dapi-stained DNA; Red, Phospho Histone 3 (PH3) stained chromosomes during mitosis; green, tubulin. The scale bar represents 5 μm. (**B**) Tubulin fluorescence images were inverted and pseudocolored so that increasing fluorescence intensity is indicated from blue to red (scale bar represent arbitrary units). Dashed lines define the region between the two spindle poles (white) and overall tubulin fluorescence within the cell (Red). (**C**) Box-and-whiskers plots show the percentage of fluorescence intensity defined by the spindle and poles in relation to the total sum of tubulin fluorescence within the cell. The boxes show the mean and the interquartile ranges, while the whiskers show the full range. (**D**) Z-stack based 2D visualization of the mitotic spindle structure in each cell following enhancement of the cytoskeletal filament networks. (**E**) Box-and-whiskers plots show the percentage of fluorescence intensity defined by the spindle and poles in relation to the total sum of microtubule fluorescence within the cell. (**F,G**) In a separate experiment cell lysates of A2780 cells were collected following 48 hours treatment with TTFields. (**F**) Immunoblot of polymerized (pol) and depolymerized (depol) tubulin fractions were compared to untreated cells and cells treated with paclitaxel or vinorelbin. (**G**) Graph represents the percentages of polymerized microtubules in relation to total tubulin. Horizontal bars indicate mean values with SD. (**H**) Immunoblot of polymerized (pol) and depolymerized (depol) tubulin fractions of cells treated with TTFields for 10 hours were compared to untreated cells, 16 hours after re-plating. (**I**) Graph represents the percentages of polymerized microtubules in relation to total tubulin. Horizontal bars indicate mean values with SD. 0.05 > *p > 0.01 and **p < 0.01 from control group.

In order to investigate whether TTFields may interfere with normal microtubule dynamics, we examined the structural properties of the mitotic spindle following TTFields exposure. A549 (lung adenocarcinoma) and MDA-MB-231 (breast adenocarcinoma; results presented in Supplementary Figure S1) cell lines were treated with TTFields (175 V/m RMS) for 24 hours at their optimal treatment frequency (150 kHz)^6^,^31^. Mitotic spindle arrangements were imaged in replicating cells during metaphase using confocal fluorescence microscopy. The results presented in Fig. 1A and Supplementary Figure S1A demonstrate that cells treated with TTFields show abnormal spindle geometry suggesting that TTFields hamper normal spindle organization. The specific anti-mitotic activity of conventional microtubule binding agents could be attributed to the fact that mitotic spindle microtubules are substantially more dynamic than the interphase cytoskeleton microtubules^34^. The spindle midzone, which is located between the two spindle poles, has been shown to exhibit the most rapid turnover in polymerization and depolymerization dynamics^35^. Therefore we calculated the ratio between the total sum of tubulin fluorescence within the cell and fluorescence intensity in the region between the two spindle poles, as described in Experimental Procedures (Fig. 1B and also in Supplementary Figure S1). The results presented in Fig. 1B,C demonstrate that in cells exposed to TTFields, the fluorescence intensity of tubulin between the two spindle poles is significantly decreased in both cell lines (see also Supplementary Figure S1). However, these results alone are insufficient to serve as a proof of abnormal microtubule polymerization, since the tubulin fluorescence intensity comprises fluorescence emission from both polymerized microtubules and the depolymerized soluble tubulin pool. Subsequently, we employed a previously reported algorithm designed to enhance cytoskeletal filament networks in order to focus our analysis specifically on microtubules^36^. The mitotic spindle structures in Fig. 1D and Supplementary Figure S1, as observed following algorithm application, clearly illustrate that when treated with TTFields, A549 and MDA-MB-231 cells exhibit severe structural mitotic spindle deformities. Furthermore, the decreased fluorescence intensity of microtubules between the two spindle poles (comprised from pole-to-kinetochore and pole-to-pole microtubules) raises the possibility that TTFields prevent spindle assembly by altering microtubule dynamics (Fig. 1E and Supplementary Figure S1). To determine whether the application of TTFields leads to a decrease in the fraction of polymerized microtubules (similar to the effects of vinca alkaloids, e.g. vinorelbine, and opposite to the effects of taxanes, e.g. paclitaxel), we compared the ratios of polymerized to total intracellular tubulin in A2780 ovarian carcinoma cells following 48 hours of exposure to 200 kHz TTFields. The fractions of free tubulin and assembled microtubules were compared to the corresponding ratios in control cells^37^,^38^,^39^,^40^. Immunoblotting for α-tubulin revealed that TTFields treated cells contained decreased levels of polymerized microtubules compared to untreated cells (12.8%, p = 0.021; Fig. 1F,G). There were no observed differences in total tubulin levels between control and TTFields treated cells (data not shown). Similar analysis conducted on U-87 MG glioblastoma cells exposed to 200 kHz TTFields reveled comparable results (Supplementary Figure S1). Microtubule turnover in dividing cells increases dramatically as cells enter mitosis^41^. This rapid turnover is especially important during this phase because it allows fast assembly of the mitotic spindle. Rapid turnover in microtubule dynamics also occurs during cell spreading^42^. Artificial conditions that encourage cell spreading (e.g. trypsinization of the cells), can be utilized to investigate extrinsic affects on microtubule polymerization and depolymerization dynamics^42^. Therefore to further examine the effect of TTFields on microtubule dynamics under rapid turnover conditions, we also measured changes in the ratio of polymerized to total tubulin that resulted from altering cell-surface contacts. For this purpose, A2780 cells were trypsinized and exposed to TTFields for 10 hours, either 6 or 30 hours after re-plating. The results in Fig. 1H,I demonstrate that cells treated with TTFields 6 hours after re-plating, (a time point at which rapid microtubule turnover rates exist), contained increased levels of depolymerized tubulin compared to untreated cells (p = 0.011). No significant change was observed between untreated cells and cells that were treated with TTFields 30 hours after re-plating, a time point at which slower microtubule turnover rates prevail (data not shown). Taken together, these results demonstrate that under rapid turnover conditions, TTFields decrease the ratio of polymerized to total tubulin, thus providing a putative explanation for the disruption of the mitotic spindle assembly apparatus.

### TTFields Treatment Results in the Formation of Multiple Nuclei within Cancer Cells

**Figure 2:**
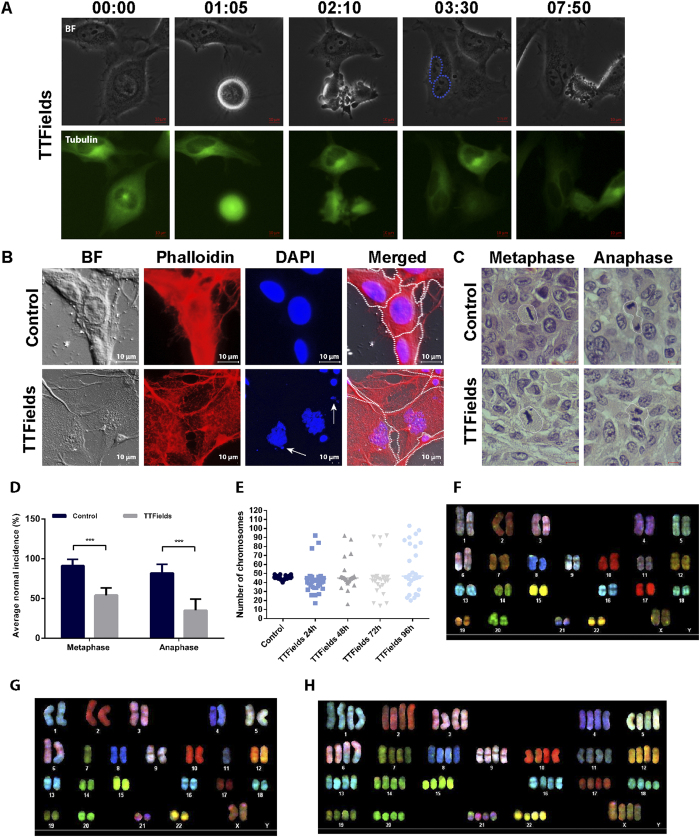
TTFields Application Induce the Formation of Multinuclear Cells and Chromosome Aneuploidy. (A) Phase-contrast and fluorescent time-lapse analysis of nuclei formation in TTFields treated Tubulin-GFP HeLa cells. Dashed line defines cell nuclei following division. Numbers show elapsed time (hour:minute). The scale bar represents 10 μm. In a separate experiment, U-87 MG cells were treated with TTFields for 72 hours. (**B**) Upper panel, U-87 MG control cells. Lower panel, TTFields treated cells. Arrow indicates micronuclei structures. Blue, Dapi-stained DNA; Red, Phalloidin-stained Actin. The scale bar represent 10 μm. Dashed line defines cell boundaries. (**C,D**) Fischer rats inoculated intracranially with F-98 glioma cells were treated with 200 kHz TTFields for 7 days, 1 week after tumor inoculation. At the end of treatment, tumors were removed and evaluated for atypical mitotic figures. (**C**) Representative images of mitotic figures. White lines encircle mitotic cells. The scale bar represent 5 μm. (**D**) Quantification of normal mitotic figures is expressed as percentage. At least 120 cells in metaphase or anaphase were analyzed in each group. ***p < 0.001 from control group. (**E**) A2780 cells were treated with TTFields for 96 hours. Chromosome number was evaluated every 24 hours. Horizontal bars indicate median values (p < 0.0001 ; Brown-Forsythe test). (**F–H**) Spectral karyotyping of A2780 cells (46, xx, der(6)t(1;6)) showing numerical aberrations following TTFields treatment. (**F**) Control.(**G**) Hypodiploidy following treatment. (**H**) Tetraploidy following treatment.

Microtubule spindle disruption was demonstrated to result in structural and numerical chromosomal abnormalities, with the latter consequently associated with multinucleated cells^43^. Therefore, we sought to examine whether TTFields exposure affects the nucleus following mitosis. Time-lapse video microscopy was utilized to directly visualize spindle structure and nuclei in the daughter cells of HeLa cervix adenocarcinoma cells expressing Tubulin-GFP (green fluorescent protein) fusion protein while exposed to 150 Hz TTFields. Although cytokinesis failure was not a common treatment outcome, long-term live cell imaging clearly revealed the disruption of the spindle structure followed by the formation of binucleate or polynucleate progeny (Fig. 2A, Supplementary Movie S1). To further explore the observation that TTFields induce multinucleation in replicating cells, we examined the behavior of other cancer cell lines exposed to TTFields. Multinuclear progeny were also formed in U-87 MG following 72 hours exposure to TTFields. The treated cells were then labeled with Phalloidin to visualize cell boundaries and were stained with the nuclear dye 4′,6-diamidino-2-phenylindole (DAPI). The results in Fig. 2B demonstrate that the formation of abnormal cells containing multiple nuclei and micronuclear structures was increased from 2% to 8% following TTFields treatment (P = 0.002). Similar results were observed for MSTO-211H mesothelioma cells where formation of abnormal cells containing multiple nuclei was increased from 1% to 18% following 150 kHz TTFields treatment (P < 0.0001) (Supplementary Figure S2). These results are in line with previous case study observation demonstrating the formation of multiple syncitial-type cells in TTFields treated glioblastoma patient, which may be the outcome of spindle disruption and abnormal chromosomes segregation^44^. Taken together, these results suggest that TTFields treatment results in nuclear aberrations including multinucleation, which is a sign of mitotic catastrophe^21^. To test whether TTFields lead to the formation of deformed mitotic figures *in vivo*, we tested their effect on Fischer rats inoculated intracranially with F-98 glioma cells. Seven days after tumor inoculation, two pairs of electrodes were attached to the rat skull. TTFields (200 V/m) were applied for 7 days using the optimal frequency of 200 kHz. Control animals were treated with temperature and geometry matched electrodes. We found that tumor volume fold increase was reduced by 41% (p = 0.05; Student’s t test) following TTFields application (n = 14) as compared to the control (n = 13), similar to the results previously published by Kirson *et al.*^6^.Histopathological analysis of treated tumors performed using hematoxylin and eosin (H&E) staining revealed a significant reduction in the percentage of cells exhibiting normal metaphase and normal anaphase (p < 0.001 for both) following TTFields application (Fig. 2C,D).

### TTFields Treatment Results in a Divergence from Normal Chromosomes Counts

Having demonstrated that exposure to TTFields leads to aberrant mitotic spindle assembly and gives rise to multinucleated progeny, we sought to analyze the effects of TTFields on cell euploidy. The chromosomally stable cell line A2780 was used for this purpose as cells are near-diploid. SKY analysis and chromosome spreads (metaphase spreads) were performed and these revealed that cells treated with TTFields show a large variation in chromosomal counts (Fig. 2E–H). There is a dramatic increase in the number of hypodiploid cells, characterized by complete chromosomal loss and this may provide a potential explanation to the observed increase in the percentage of resultant micronuclear structures. Of note, we also conducted Giemsa staining analysis, the results of which excluded the possibility of partial chromosomal loss (data not shown). Comparable results were also obtained in U-87 MG (Supplementary Figure S2) and AsPC-1 pancreatic adenocarcinoma cell lines following 150 kHz TTFields treatment (Supplementary Figure S2).

### Variations in Response to TTFields are Independent of Model Chromosome Number and can be Explained by Differences in Cell Doubling Time

**Figure 3:**
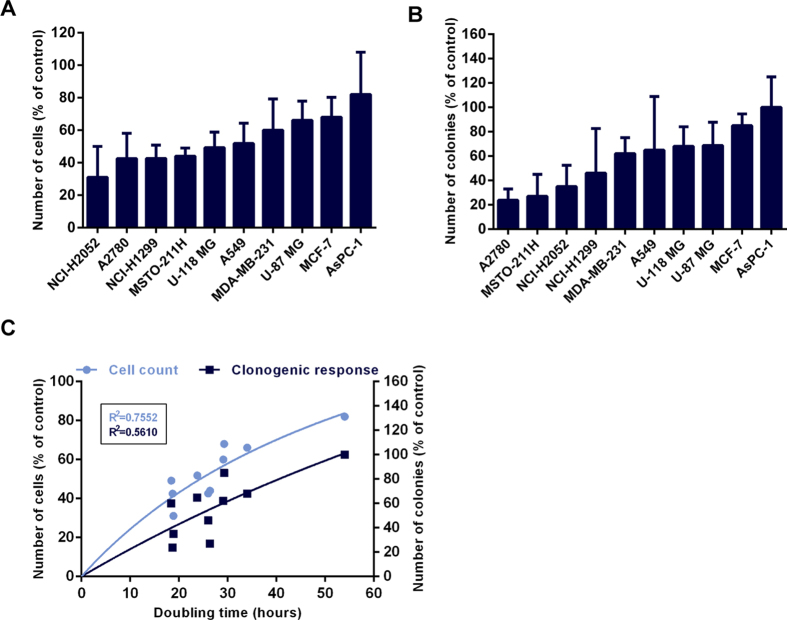
TTFields Treatment Efficacy Is Cell Doubling Time Dependent. Comparison of cell lines response to TTFields treatment. Cells were treated with TTFields for 72 hours at optimal frequency (Table 1). (A) Effect of TTFields treatment on cell count of various cancer cell lines. (**B**) Clonogenic survival of various cancer cell lines following TTFields treatment. (**C**) Correlation between cell doubling time and cell count (p = 0.008) and clonogenic response (p = 0.023) following TTFields treatment.

Understanding how abnormal chromosome dynamics affect cell viability could be critical to uncovering why some cancer cells are more susceptible to the effects of TTFields than others^6^,^7^,^31^. Missegregation of chromosomes may result in rapid cell death, since a minimum number of chromosomes are requisite for cellular viability^46^. We systematically compared the changes in cell count and clonogenic survival in ten solid-tumor derived cell lines following 72 hours of TTFields treatment. All tested cell lines, demonstrated reduction in cell count (Fig. 3A) as well as reduced clonogenic survival (Fig. 3B) following the application of TTFields. However, there was no clear correlation between model chromosome number and clonogenic survival (Spearman rank correlation coefficient, data not shown). For example, although the hypertriploid MCF-7 breast adenocarcinoma exhibited a relatively poor response to TTFields exposure, other hypertriploid cells such as MSTO-211H demonstrated a substantial decrease in both cell count and clonogenic capacity following TTFields treatment. We tested the hypothesis that cells which present with a hypodiploid karyotype prior to treatment may be more susceptible to TTFields treatment. We observed that the hypodiploid malignant glioma cell lines U-87 MG and U-118 MG did not exhibit higher sensitivity to TTFields, as compared with cells with a higher chromosome number at baseline e.g. A549. Based on these data, we concluded that there is no correlation between pre-treatment karyotype and treatment outcome. As TTFields disrupt normal mitosis, the correlation between treatment efficacy and treated cell doubling time was also evaluated. A significant positive Spearman rank correlation was found between treated cell doubling time and both cell count (r = 0.7939; p = 0.008) and clonogenic survival (r = 0.7212; p = 0.023) following TTFields exposure (Fig. 3C). Together, these results demonstrate that within any given treatment period, rapidly dividing cells are more likely to be affected by TTFields while non dividing cells remain intact.

### TTFields Efficacy can be Enhanced by Extending Treatment Duration

**Figure 4:**
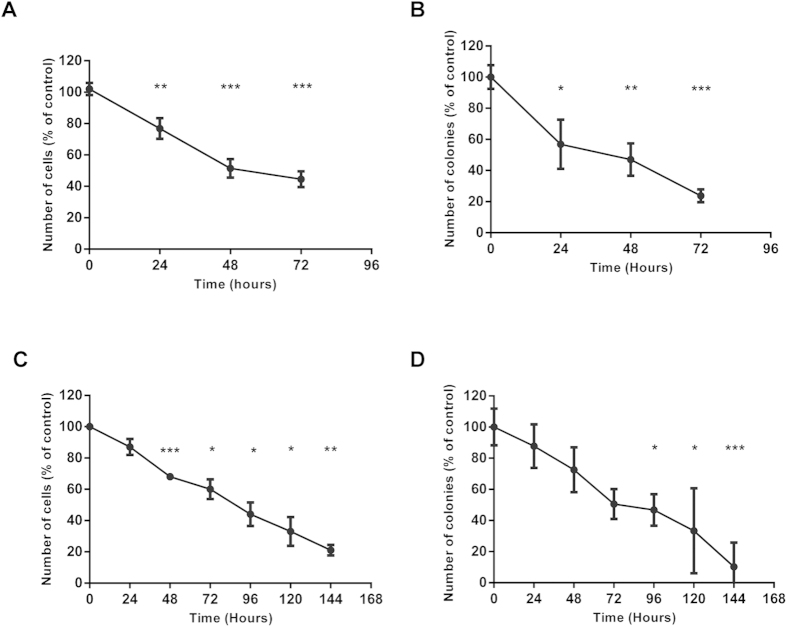
TTFields Treatment Efficacy is Dependent on Treatment Duration. (**A,B**) A2780 cells were treated with TTFields for 72 hours. (**A**) Effect of TTFields treatment on number of A2780 cells. (**B**) Clonogenic survival of A2780 cells following TTFields treatment. (**C,D**) In a separate experiment, U-87 MG cells were treated with TTFields for 144 hours. (**C**) Effect of TTFields treatment on number of U-87 MG cells. (**D**) Clonogenic survival of U-87 MG cells following TTFields treatment. 0.05 > *p > 0.01, **p < 0.01, and ***p < 0.001 from control group.

The observation that cell doubling time can determine response to TTFields led us to explore the impact of varying the duration of treatment exposure. The results presented in Fig. 4A,B show that increasing the duration of treatment improves cytotoxic effects in A2780 cells (doubling time – 18.7 hours) as shown by a decrease in both cell count and clonogenic survival. In order to exclude the possibility that this augmented effect was detected by virtue of the longer observation period (as opposed to an absolute improvement in efficacy), we also preformed a release assay in which A2780 cells were treated for limited time intervals (24, 48, 72 hours) and cell count was monitored in subsequent time frames (e.g. for 24 hours treatment, 48 and 72 hours). The results in Supplementary Figure S3 demonstrate that for longer treatment periods the decrease in the percentage of viable cells was greater than that achieved with short treatment periods followed by longer observation timeframes. Therefore, although long-term effects of TTFields are maintained after a treatment break, extending the duration of TTFields application resulted in improved treatment outcomes. We next examined the possibility of improving efficacy for cells possessing longer doubling times. TTFields were applied for longer periods in U-87 MG (doubling time – 39 hours) and AsPC-1 (doubling time – 54 hours) cells. A longer period of exposure to TTFields resulted in a significant decrease in both cell count and clonogenic survival in U-87 MG cells (Fig. 4C,D). Comparable results demonstrating enhanced efficacy achieved by increasing treatment duration were also obtained for the AsPC-1 cell line (data not shown). These observations are consistent with the effect of TTFields on the mitotic spindle and suggest that TTFields treatment efficacy is amplified when cells undergo additional mitosis during treatment exposure. Overall, these results reinforce our hypothesis that TTFields treatment exclusively affects dividing cells.

### TTFields Exposure Cause Both Mitotic Cell Death and Death in Interphase

**Figure 5:**
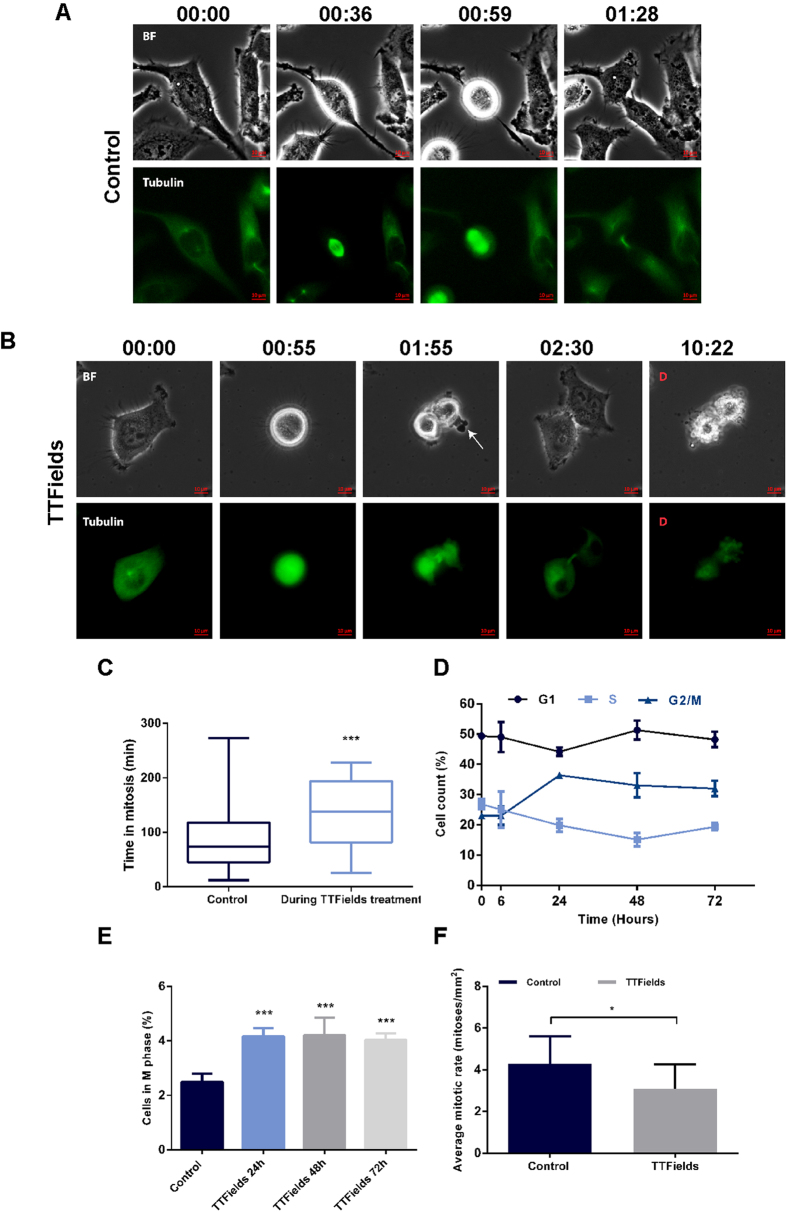
Mitotic Arrest and Mitotic Cell Death Are Possible Outcomes of TTFields Treatment. (**A–C**) Phase-contrast and fluorescent time-lapse analysis of mitosis in Tubulin-GFP HeLa cells. (**A**) Control cells. (**B**) TTFields treated cells (8 h). Numbers show elapsed time relative to entry to mitosis (hour: minute). Arrow indicates membrane blebbing. Cell death is indicated as D. The scale bar represents 10 μm. (**C**) Box-and-whiskers plots show the time that cells spent arrested in mitosis. The boxes show the mean and the interquartile ranges, while the whiskers show the full range. (**D,E**) In a separate experiment, A2780 cells were treated with TTFields for 72 hours. (**D**) Cell cycle analysis was performed using flow cytometry. (**E**) Graph represents the change in percentage of A2780 cells in M phase following TTFields application. (**F**) Fischer rats inoculated intracranially with F-98 glioma cells were treated with 200 kHz TTFields for 7 days 1 week after tumor inoculation. At the end of treatment, tumors were removed and evaluated for average mitotic rate. 0.05 > *p > 0.01, ***p < 0.001 from control group.

Our data support a model in which TTFields disrupt mitotic spindle morphology, leading to chromosome missegregation and subsequent aneuploidy. However the fate of the affected cells is unknown. Time-lapse video microscopy was used to establish whether TTFields exposure to HeLa cells expressing Tubulin-GFP fusion protein ultimately activates a death pathway while cells are still in mitosis (mitotic cell death) or whether cell death is activated in the subsequent interphase period. Results demonstrated that TTFields treated cells exhibited a significant yet short increase in mitotic duration as compared to controls (132.4 ± 60.8, 86.7 ± 51.1 minutes, respectively, p < 0.001) (Fig. 5A–C). Follow-up observations for the resulting progeny revealed that one or both daughter cells underwent cell death (67% of mitotic cells) within 21.5 ± 19.4 hours following mitosis (Supplementary Movie S2). Of total 75% failed cell divisions, 8% of deaths occurred while in mitosis. To determine whether TTFields induce mitotic arrest in other cancer cell lines, we performed cell cycle analysis using flow cytometry and examined the relative fraction of cells in G2/M phase across various cell lines. The results in Fig. 5D, demonstrated that A2780 cells exposed to TTFields exhibited accumulation in G2/M (32.0 ± 2.6% after 72 hours treatment relative to 23.0 ± 0.9% in control, p < 0.01). We further tested the accumulation of A2780 cells specifically in M phase, using anti-phosphorylated (ser10) H3 in order to discriminate between cells in G2 and M phase^47^. The results in Fig. 5E showed that the number of A2780 cells accumulating in M-phase significantly increased from baseline following exposure to TTFields, similar to our time-lapse observations from HeLa cells. In contrast, U-87 MG cell accumulation was observed to peak in G1 phase at 48 hours (data not shown). It is possible, that while in certain cells, disruption of the spindle structure by TTFields may increase mitosis duration, in other cells these perturbations may result in reduced mitotic rate. Indeed, similar to the reduced proliferation in the U-87 MG cells, a significant reduction (p < 0.02) in the mitotic rate was also observed in the rat F98 glioma model following 7 days of treatment with TTFields (Fig. 5F).

To determine whether the growing fraction of cells in G1 were senescent cells, we performed a β-galactosidase senescence assay. There was no evidence to suggest a difference in senescence phenotype between untreated and U-87 MG cells treated with TTFields for 24 or 120 hours (data not shown). To further address the possibility of induction of senescence, AsPC-1, H-1299 lung carcinoma, MCF-7, MDA-MB-231, A2780, and A549 cells were also examined for β-galactosidase activity. There was no evidence to suggest exposure to TTFields promoted cellular senescence following 48 hours of TTFields treatment in these cell lines (data not shown). Overall, our data suggest that TTFields treated cells exhibit small delays in mitosis following which some cells die in mitosis while others eventually divide and continue to the next interphase. In totality, these results suggest there is probably more than a singular cell fate following TTFields exposure.

### TTFields Exposure Induces Caspase-Mediated Cell Death

**Figure 6:**
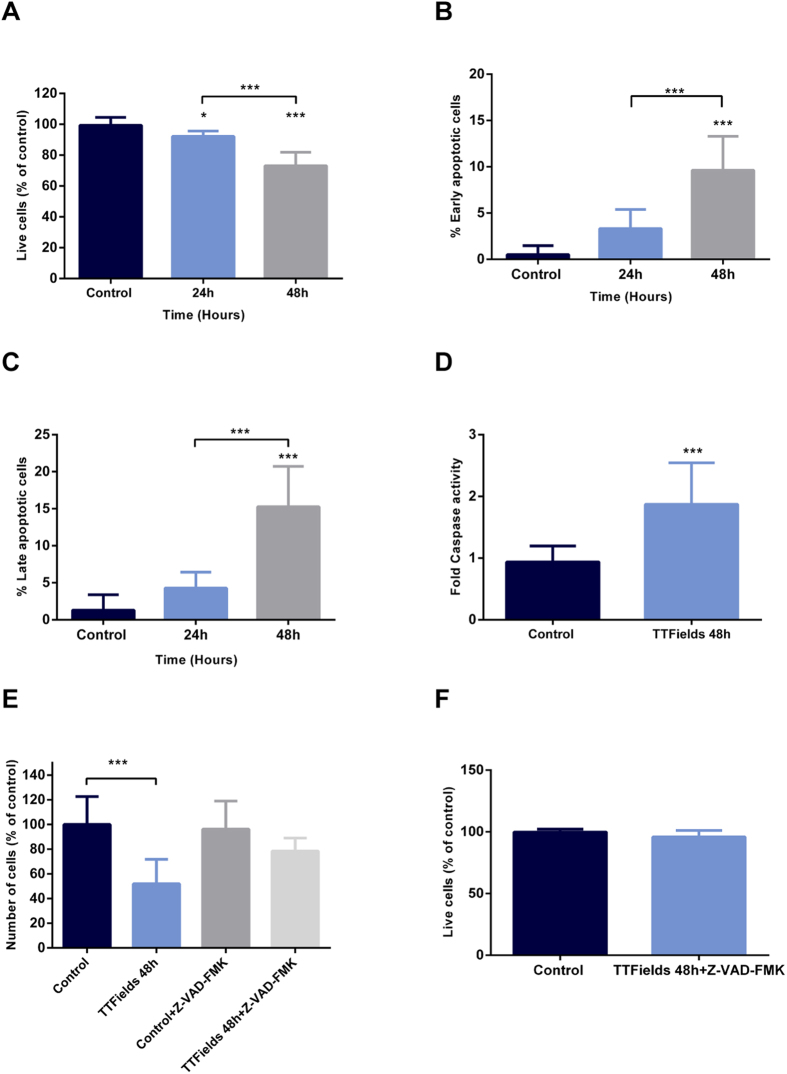
TTFields Kill Cancer Cells by Triggering Caspase Mediated Apoptosis. A2780 cells were treated with TTFields for 48 hours. (**A–C**) Cell samples were collected and evaluated for apoptosis. (**A**) Live cells. (**B**) Early apoptotic cells. (**C**) Late apoptotic cells. (**D**) Caspase activity was evaluated using flow cytometry analysis following 48 hours of TTFields application. (**E,F**) Effect of pan-caspase inhibition by Z-VAD-FMK on A2780 response to TTFields treatment. (**E**) Evaluation of number of cells (unpaired t-test). (**F**) Evaluation of apoptosis. 0.05 > *p > 0.01, and ***p < 0.001 from control group.

The correlation between doubling time, cell count and clonogenic survival following TTFields exposure suggests that maximal cytotoxic effects may only manifest after most of the cell population has completed the first interphase while receiving treatment. This hypothesis is supported by our time-lapse observations where the majority of the cells died following completion of mitosis (second interphase). To test this hypothesis we performed flow cytometry analysis on A2780 cells at different time points during TTFields treatment, using staining for Annexin V and Propidium Iodide (PI) for detection of early (Annexin V+/PI−) and late (Annexin V+/PI+) apoptosis, respectively. The results in Fig. 6A–C demonstrate that the percentage of cells undergoing early and late apoptosis dramatically increased following TTFields treatment. To determine whether the apoptosis was caspase dependent we used a caspase detection fluorescent probe to examine caspase activity in cells following 48 hours of TTFields treatment. Compared with control cells, treatment of A2780 cells with TTFields led to a 1.9 fold increase in caspase activity (Fig. 6D). To determine the extent to which caspase dependent apoptosis accounts for the cytotoxic effects of TTFields, we tested the changes in cell count following TTFields treatment in the presence of the broad-spectrum caspase inhibitor, benzyloxycarbonyl-Val-Ala-Asp-fluoromethyl ketone (Z-VAD-FMK). Treatment of cells with the caspase inhibitor severely impaired cytotoxicity induced by TTFields treatment. While the average cell count of A2780 cells treated with TTFields for 48 hours was 52 percent, in the presence of Z-VAD- FMK cell count rose to 79 percent (Fig. 6E). Of significance, apoptosis was completely abrogated in the presence of Z-VAD-FMK confirming that TTFields induced cell death was caspase dependent (Fig. 6F). Comparable results were obtained for U-87 MG cells (Supplementary Figure S4).

## Discussion

Our results provide the first evidence supporting the direct effect of TTFields on microtubules, and specific effects on spindle assembly in replicating cells. We show for the first time, to our knowledge, that TTFields destabilize microtubules. Whether this effect is achieved by direct interference of the addition of tubulin subunits to microtubules, or by way of destruction of existing microtubule structures, remains to be determined. Increased percentages of depolymerized tubulin in cells treated with TTFields while spreading following re-plating supports the possibility that TTFields interfere with polymerization of microtubules^42^. Higher resolution studies should help further elucidate these effects.

Other investigators have found that a similar extent of alteration in the ratio of polymerized to total intracellular tubulin, (as is observed with TTFields application), can lead to paclitaxel resistance^48^. Interestingly, our prior investigations conversely suggested that TTFields treatment improves paclitaxel treatment efficacy^31^,^49^. These conflicting observations suggest that there may be additive efficacy in combining TTFields with spindle poisons, a notion which is supported by clinical observations in which the combination of vinorelbine with paclitaxel in metastatic breast cancer patients has been associated with superior antitumoral activity^50^.

TTFields frequencies utilized in this series of investigation are the specific frequencies that have led to the highest reduction in cell counts, most likely by virtue of their effect on the mitotic spindle. In addition to mitosis, there are other biological conditions where rapid turnover in microtubule dynamics could be affected by TTFields. Especially relevant are cellular invasion and migration in which inhibition of microtubule formation could result in alternation of a cell’s metastatic potential^51^,^52^. It is possible that TTFields in frequencies other than those used in this study could influence cellular motility, invasion and migration.

**Figure 7:**
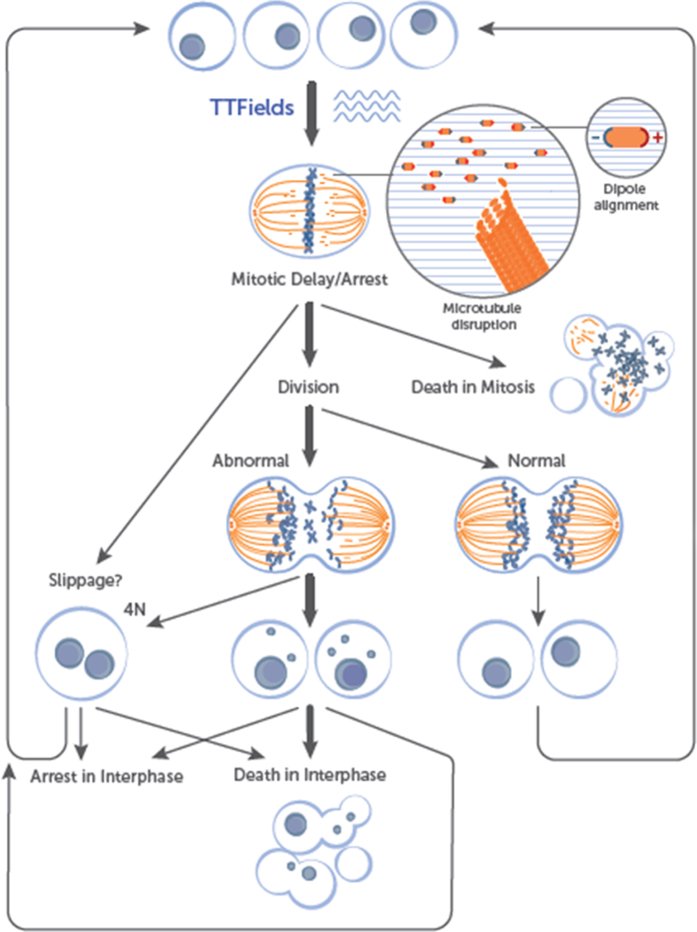
Effects of TTFields on replicating cells. TTFields exert directional forces on polar microtubules and interfere with the assembly of the normal mitotic spindle. Such interference with microtubule dynamics results in abnormal spindle formation and subsequent mitotic arrest or delay, possibly due to improper attachment of chromosomes to the spindle fibers. Cells can die while in mitotic arrest, however, a more common outcome (highlighted by bold arrow) is progression to cell division. This can lead to the formation of either normal or abnormal aneuploid progeny. The formation of the tetraploid cells can occur either due to mitotic exit through slippage or can occur during improper cell division. Abnormal daughter cells can die in the subsequent interphase, can undergo a permanent arrest, or can proliferate through additional mitosis where they will be subjected to further TTFields assault.

Our findings highlight important insights regarding cell fate following spindle disruption by TTFields (Fig. 7). While prolonged mitotic arrest can occur following TTFields exposure, cell death during the subsequent interphase is more prevalent. Although the initial triggers of caspase dependent apoptosis that arise following TTFields treatment are not fully understood, our data strongly suggest that, in addition to mitotic arrest, an accumulation of massive aneuploidy contributes to this compromised viability. The notion that a microtubule- targeting modality can lead to aneuploidy and subsequent cell death is supported by recent results by Zasadil *et al.* demonstrating that paclitaxel treatment leads to cell death in patients by inducing chromosome missegregation without mitotic arrest^53^. Aneuploidy has long been argued to drive tumorigenesis and promote tumor progression^54^,^55^,^56^,^57^. However, there is now an expanding body of evidence suggesting that chromosome missegregation can also be an inhibitor of tumorigenesis^56^,^58^,^59^,^60^. Silk *et al.* have recently suggested that levels of aneuploidy elevated beyond a certain threshold, suppress tumors by causing cell death^46^. Thereby, it can be argued that acceleration of massive chromosome missegregation is a useful therapeutic strategy. It remains unclear, however, whether TTFields induced post mitotic cell death is a sole outcome of aneuploidy in subsequent interphase or whether it is also a delayed manifestation of cellular damage which occurs during mitosis. Our results suggest that TTFields induced cell death occurs several hours following completion of mitosis. Thus, a post mitotic response which involves activation of the p53 pathway is more likely^61^,^62^. The influence of p53 status on variation in response to TTFields therapy is currently being investigated.

In addition, our time lapse microscopy and cell cycle data suggest that there is probably more than a singular cell fate following TTFields exposure. These observations are in line with growing body of evidence suggesting both inter and intra–line variation in response to anti-mitotic drugs^17^,^63^,^64^. We do not have a clear explanation to account for these divergences in cell fate. It is possible that while completion of cell cytokinesis is prevalent in TTFields treated HeLa cells, mitotic arrest and cell death arising directly from mitosis could be a significant response to TTFields exposure in other cell lines. Differences in mitotic spindle, SAC status, and differences in apoptotic signaling could all be factors in determining whether or not, and to what extent, mitotic cell death is attained^26^,^65^.

Our results also provide a potential explanation as to why cell lines respond differently to TTFields, and offer suggestions for obtaining improvements in therapeutic responses. As the mechanism of action of TTFields involves disruption of spindle microtubules, consequently leading to mitotic catastrophe, cells entering mitosis are those most likely to respond to TTFields. Our observations suggest that *in vitro* treatment duration should therefore vary between cell lines and be in accordance with their cell doubling time, in order to allow a maximal fraction of cell population to pass through mitosis. Extension of treatment duration proved to enhance treatment efficacy as gradual decreases in both cell viability and clonogenic survival were observed as treatment continued. It is reasonable to assume that the progeny of cells which succeeded in completing previous mitosis under TTFields treatment were further damaged on the consecutive mitotic events as the treatment duration was extended. This conclusion is supported by clinical observations where overall survival outcomes are more favorable in those patients with recurrent glioblastoma who maintain higher therapeutic compliance with TTFields therapy^10^. Future randomized clinical trials will however be necessary to confirm this, as clinical response to taxanes, for example, which also target mitotic cells, does not correlate with tumor proliferation rate^66^. It should be acknowledged, however, that the effective doses of systemic drugs vary greatly and are dependent on pharmacokinetic and pharmacodynamic factors such as plasma concentration and elimination half life^17^. In this context, the application of TTFields presumably resembles a state in which constant drug concentration is delivered to the tumor. Therefore, it is likely that data obtained from cell culture studies might indeed advance our understanding of *in vivo* response to TTFields, ultimately enhancing clinical trial designs to improve patient outcomes.

## Methods

### Cell culture and drugs

Human ovarian carcinoma cell line A2780 was obtained from the European Collection of Cell Cultures (ECACC). All other cell lines were obtained from the American Tissue Culture Collection (ATCC). Human lung adenocarcinoma cell lines H1299 and A549, human pancreatic adenocarcinoma cell line AsPC-1, human ovarian carcinoma cell line A2780, and human mesothelioma cell lines NCI-H2052 and MSTO-211H, were grown in RPMI Medium. Human glioblastoma cell line U-87 MG, human cervical adenocarcinoma cell line HeLa, and human breast adenocarcinoma cell line MCF-7, were grown in Eagle's Minimum Essential Medium. Human glioblastoma cell line U-118 MG, human breast adenocarcinoma cell line MDA-MB-231, and rat glioblastoma cell line F98 were grown in Dulbecco’s modified Eagle’s medium. All culture media were supplemented with 10% (v/v) fetal bovine serum, 2 mM L-glutamin and penicillin/streptomycin (50 μg/ml). All cells were incubated in a humidified incubator supplied with 5% CO_2_. Electrical parameters of media are provided in Supplementary Table S1. Differences within the range of 2-6% between the average resistivity and current density of the different media were measured and considered to be negligible. Small molecules dissolved in DMSO were used at the following concentrations unless stated otherwise: paclitaxel (Sigma) 33 nM, benzyloxycarbonyl-Val-Ala-Asp-fluoromethyl ketone (Z-VAD- FMK) (Promega) 20 μM. Vinorelbine (Sigma Aldrich) dissolved in water was used at 30 nM.

### TTFields application *in vitro*

TTFields were applied as previously described^31^ using the inovitro system. Briefly, two pairs of transducer arrays were printed perpendicularly on the outer walls of a Petri dish composed of high dielectric constant ceramic [lead magnesium niobate–lead titanate (PMN-PT)]. The transducer arrays were connected to a sinusoidal waveform generator which generated fields at the desired frequencies in the medium as summarized in Table 1. The orientation of the TTFields switched 90° every 1 second, thus covering the majority of the orientation axis of cell divisions, as previously described by Kirson *et al.*^7^. Temperature was measured by 2 thermistors (Omega Engineering, Stamford, CT) attached to the ceramic walls. All cells suspensions were grown on a cover slip inside the inovitro dish and treated with TTFields at intensity of 175 V/m. TTFields were applied for the time frames indicated in figure legends.

### Tumor inoculation and *in vivo* size assessment

All animal studies were approved by the Technion–Israel Institute of Technology institutional animal care and use committee, and were performed in accordance with the committee’s relevant guidelines and regulations. F-98 glioma cells were inoculated stereotactically into the subcortical white matter in the right hemisphere of Fischer rats (Harlan laboratories, Israel) by using a modification of the method previously described^67^,^68^. Briefly, a hole, 3 mm in diameter, was punched through the scalp, 2 mm to the right of the midline and 4 mm rostral to the line connecting the external ear canals. A 1.0 mm burr hole was drilled in the bone at same location and a 26G needle was inserted to a depth of 7 mm beneath the scalp surface. Five microliters of saline containing 1x 10^4^ F-98 cells mixed with 50% high-concentration growth-factor–reduced Matrigel (BD Biosciences, Bedford, MA) were then injected by using a microsyringe operated by a micromanipulator. The needle was left in position for 20 sec and then retracted slowly at a rate of 2 mm/min. Rats were allowed to recuperate for 7 days before treatment initiation. Tumor volume was assessed based on serial (1-mm interval) T1 weighted axial MRI images (7T MRI scanner; Bruker, Karlsruhe, Germany) obtained 5 min following injection of 0.4 ml of Gadolinium-DTPA (0.125 mM) (Magnetol; Soreq Radiopharmaceuticals, Yavne, Israel) into the tail vein. The protocol included one T1 weighted image pre-GD injection and one post-GD injections T1 weighted image. Tumor volume was assessed by calculating the area in square millimeters of the contrast enhanced lesion in each section.

### TTFields application *in vivo*

Application of TTFields (200 kHz) to the rat brain was initiated 7 days after intracranial tumor inoculation and was maintained for 7 days. Two pairs of electrodes, each composed of 2 disks with a radius of 3 mm, were attached to the rat skull in dorsolateral and left-right positions generating two different field directions. The capacitance of each disk was about 30 nF. Each disk contained a thermistor in order to allow for constant temperature monitoring. The current source output was switched, every 1 s, between the 2 electrodes. Control rats were treated by means of sham electrodes which were geometrically matched to the TTFields group. The Sham heat electrodes produced equal temperature changes to those produced by the field electrodes by means of a heating resistor incorporated within them. Each rat was placed inside a separate cage and the electrodes were connected to the NovoTTF-100A device. Rats were checked twice daily for their physical condition. At the end of treatment, electrodes were removed and the tumor volume was determined as described. The rats were euthanized and the tumors were removed.

### Classification of mitotic disturbances and mitotic index in tumors

F-98 tumor formalin-fixed, paraffin-embedded sections (10 μm) were stained with hematoxylin and eosin (H&E). All available slides were reviewed by independent pathologist (Ori Brenner, DACVP), and average mitotic rate was blindly evaluated. Atypical mitotic figures were determined in a blinded fashion based on intact or chromosome configurations during metaphase and anaphase. At least 120 cells in metaphase or anaphase were analyzed in each group.

### Tubulin polymerization assay

Tubulin polymerization was determined as previously described^40^. In brief, cells were harvested with lysis buffer (2 mM EGTA, 1 mM MgCl2, 0.5% NP40, 2 mM phenylmethylsulfonyl fluoride, 20 mM Tris HCl pH6.8) containing protease inhibitors (Sigma-Aldrich). The samples were then centrifuged at 13,000 × g for 10 min at 22 °C. The supernatant (containing depolymerized tubulin) was transferred to a new tube and the pellet (polymerized microtubules) was resuspended in an equal volume of lysis buffer. For controls, cells were cultured with either paclitaxel (16 hours) or vinorelbine (A2780, 6 hours; U-87 MG, 16 hours). Protein fractions were then immunobloted for further analysis.

### Western blot

Laemmli sample buffer (×4) (Bio-Rad) was added to 30 μg of each protein sample and the mixtures were boiled at 95 °C for 5 minutes. Proteins were then loaded on 7.5% SDS-PAGE gel (Bio-Rad) followed by transfer to nitrocellulose membrane for 1 hour at 100 V, 4˚C. The membrane was then blocked with 5% milk in TBST for 1 hour and probed with rabbit anti-α-tubulin (0.5 μg/ml) (Abcam) overnight at 4 °C. The membrane was further probed with goat anti-rabbit conjugated with horseradish peroxidase (1:10,000) (Jackson immunoresearch) for 1 hour. Bands were detected using chemiluminescence detection kit (Biological Industries), and quantification was performed by densitometry. The percentage change in depolymerized tubulin was determined by the following equation: (gained densitometric values of depolymerized tubulin/sum of gained densitometric values of polymerized and depolymerized tubulin) × 100.

### Microscopy

For spindle structure analysis, cells were grown on glass cover slips and treated using the inovitro system for 24 hours. At the end of the experiment, cells were fixed with ice cold methanol for 1 hour at −20 °C. The cells were then serum-blocked, and stained with mouse anti-human pH3 and rabbit anti-human α-tubulin antibodies (Abcam) overnight at 4 °C. Alexa Fluor–conjugated secondary antibodies were used (Abcam). DNA was stained with the dye 4′,6-diamidino-2-phenylindole (DAPI) (Sigma-Aldrich) at 0.2 μg/ml for 20 min. Images were collected using a LSM 700 laser scanning confocal system, attached to an upright motorized microscope with a × 63/1.40 oil objective (Zeiss Axio Imager Z2). Reconstruction of Z-stack images was performed using ZEN 2012 lite (Zeiss) software.

For multinucleation analysis, cells were grown on glass cover slips and treated using the inovitro system for 72 hours. At the end of the experiment, cells were fixed with freshly prepared 4% paraformaldehyde for 10 min at room temperature and permeabilized with 0.1% Triton X-100. Cells were then stained at room temperature with 50 μg/ml phalloidin (Sigma-Aldrich) for 40 min and with DAPI. Images were collected on a Zeiss Axio observer inverted motorized fluorescent microscope equipped with a high sensitivity B/W fluorescent camera (Hamamatsu Orca R2).

### Spindle structure image processing

To estimate the fraction of microtubules polymerized in the spindle, 3D confocal stacks of cells in which the tubulin was fluorescently labeled were taken, and the ratio between the fluorescence intensity in the region between the two spindle poles (pole-to-kinetochore and pole-to-pole tubulin, also include the spindle midzone) and the total fluorescence intensity within the cell was calculated for each imaged cell. This was done using a semi-automatic procedure realized in Matlab™ 2010. First the volumes of entire cells were manually identified in the confocal stacks. This was followed by manual segmentation of the volume confined by the mitotic spindle and the two spindle poles within each cell. Finally, the ratio between the total intensity within the region defined by the spindle and poles, and the total intensity within the entire cell was calculated. For assessment of microtubule organization, a previously reported algorithm designed to enhance cytoskeletal filament networks was used^36^. This algorithm comprises the following steps: First background is subtracted using a morphological top-hat filter, followed by histogram equalization. Next, a steerable ridge detector is used to enhance microtubule structures. This is followed by filament thinning using non-maximum suppression. The end result is an image in which fibers with a width of a single pixel are clearly visible. The algorithm was applied to each confocal slice separately, followed by creation of a maximum projection to yield a 2D visualization of the microtubule structure in each cell. The algorithm was realized using Matlab™ R2010. Steerable filtering was performed using Matlab code written by C. Yuan, which is available for download at:

[ http://www.mathworks.com/matlabcentral/fileexchange/30253-blood-cells-tracking-and-measurement-by-using-spatiotemporal-images-analysis]. Non-maximum suppression was performed using Matlab code written by P. Kovesi:

[ http://www.csse.uwa.edu.au/~pk/research/matlabfns/].

### Time-lapse imaging

For Time-lapse video microscopy, GFP-tagged tubulin was introduced into HeLa cells using the CellLight BacMam 2.0 kit as per manufacturer instructions (Molecular Probes). HeLa cells expressing Tubulin-GFP fusion protein were then imaged during 8 hours of TTFields application following which image acquisition was continued for a further 40 hours. TTFields were applied as described previously^69^. Imaging was performed using an inverted motorized fluorescent microscope (Zeiss Axio observer) equipped with a stage incubator (Live Imaging Services) and a high resolution B/W fluorescent camera (Zeiss Hrm). Movies were assembled using ZEN 2012 lite (Zeiss) software.

### Flow Cytometry

For cell cycle analysis, cells were washed twice with PBS and fixed with cold 70% ethanol. After incubation with 10 μg/ml RNase and 50 μg/ml propidium iodide (PI) (Sigma-Aldrich) at 37 °C for 30 minutes. For detection of apoptotic cells 1 × 10^6^ cells were double stained with CF633-conjugated annexin V (Biotium) and PI for 15 min at room temperature. Binding buffer was added before analysis. For detection of caspase activity, cells were stained using FAM FLICA poly caspase assay kit (Immunochemistry technologies) as per manufacturer instructions. Data acquisition was obtained using BD LSR Fortessa (BD Biosciences) flow cytometer. Fluorescence signals were collected at the wavelengths of 610/20 nm for PI, 670/14 nm for annexin V, and 530/30 nm for FLICA. The data was analyzed using the FACSDiva software.

### Chromosome Analysis

SKY was performed using SKY Paint kit-Human (Applied Spectral Imaging) as per manufacturer’s protocol. The samples were then analyzed using the D300 bio imaging system (Applied Spectral Imaging). For chromosome spreading, cultured cells were treated with Colchicin (10 μg/ml for 6 h). Cells were then collected, and the suspended cells were treated with hypotonic solution (KCl) and fixed with Methanol and Acetic Acid (3:1 ratio). Chromosome spreading and Giemsa staining (4% in Gurr buffer for 2 h) (Gibco) were performed by conventional methods.

### Cell count

Inhibition of tumor cell growth was analyzed by quantitatively determining cell count using Scepter 2.0 automated cell counter (EMD Millipore). The relative number of cells at the end of treatment was expressed as percentage of untreated control.

### Clonogenic survival assay

Cells treated with TTFields were subsequently harvested and re-plated into 6-well tissue culture plates (300 cells/well). Five to 10 days after seeding, colonies were stained with 0.5% crystal violet, and the number of colonies containing at least 50 cells was counted. Survival fractions were calculated relative to control.

### β-Gal assays

Detection of β-gal activity was performed using Senescence Cells Histochemical Staining Kit (Sigma-Aldrich) as per manufacturer instructions.

### Statistical analysis

Unless stated otherwise, data are expressed as mean ± SD, and the statistical significance of differences was assessed by 1-way ANOVA followed by Tukey’s range statistical test using GraphPad Prism 6 software. Differences between all groups were compared with each other, and were considered significant at values of 0.05 > *p > 0.01, **p < 0.01, and ***p < 0.001. Correlation between cell doubling time and cell count and clonogenic survival was estimated using Spearman’s rank correlation coefficients.

## Additional Information

**How to cite this article**: Giladi, M. *et al.* Mitotic Spindle Disruption by Alternating Electric Fields Leads to Improper Chromosome Segregation and Mitotic Catastrophe in Cancer Cells. *Sci. Rep.* **5**, 18046; doi: 10.1038/srep18046 (2015).

## Supplementary Material

Supplementary Information

Supplementary Movie S1

Supplementary Movie S2

## Figures and Tables

**Table 1:** Summary of cell line specific features that were taken in consideration for this study.

**Cell line name**	**Tissue**	**Disease**	Karyotype*	**Optimal frequency (kHz)**	**Doubling time (hours)**
A2780	Ovary	Carcinoma	Modal chromosome number = 46	200	18.7
A549	Lung	Adenocarcinoma	Hypotriploid, modal chromosome number = 66 in 24% of cells	150	23.8
AsPC-1	Pancreas	Adenocarcinoma	Not specified	150	54.0
HeLa	Cervix	Adenocarcinoma	Modal number = 82; range = 70 to 164	150	24
MCF-7	Mammary gland; breast	Adenocarcinoma	Hypertriploidy to hypotetraploidy, modal chromosome number = 82	150	29.3
MDA-MB-231	Mammary gland: breast	Adenocarcinoma	Near-triploid, modal number = 64	150	29.1
MSTO- 211H	Lung	Biphasic mesothelioma	Modal chromosome number = 72	150	26.4
NCI-H1299	Lung	Carcinoma; NSCLC	Not specified	150	23.1
NCI-H2052	Lung	Stage 4, mesothelioma	Not specified	200	18.9
U-87 MG	Brain	Grade IV glioblastoma; astrocytoma	Hypodiploid, modal chromosome number = 44 in 48% of cells	200	34.0
U-118 MG	Brain	Grade IV glioblastoma; astrocytoma	Hypodiploid, modal chromosome number = 44 in 48% of cells	200	18.5

^*^According to ATCC and/or NCI SKY/M-FISH & CGH Database.
