# Supplementary material for: Mitotic Spindle Disruption by Alternating Electric Fields Leads to Improper Chromosome Segregation and Mitotic Catastrophe in Cancer Cells
Source: Sci Rep. 2015 Dec 11;5:18046. doi: 10.1038/srep18046 (PMC4676010; doi:10.1038/srep18046)
Supplement: Supplementary Information [file srep18046-s1.pdf]

Supplementary Information

Mitotic Spindle Disruption by Alternating Electric Fields Leads to Improper Chromosome Segregation and Mitotic Catastrophe in Cancer Cells

Moshe Giladi[1,2], Rosa S Schneiderman[1,2], Tali Voloshin[1], Yaara Porat[1], Mijal Munster[1], Roni Blat[1], Shay Sherbo[1], Zeev Bomzon[1], Noa Urman[1], Aviran Itzhaki[1], Shay Cahal[1], Anna Shteingauz[1], Aafia Chaudhry[1], Eilon D Kirson[1], Uri Weinberg[1], and Yoram Palti[1]

[1]Novocure Ltd.
Topaz Building, MATAM center
Haifa 31905, Israel
[2]These authors contributed equally to this work

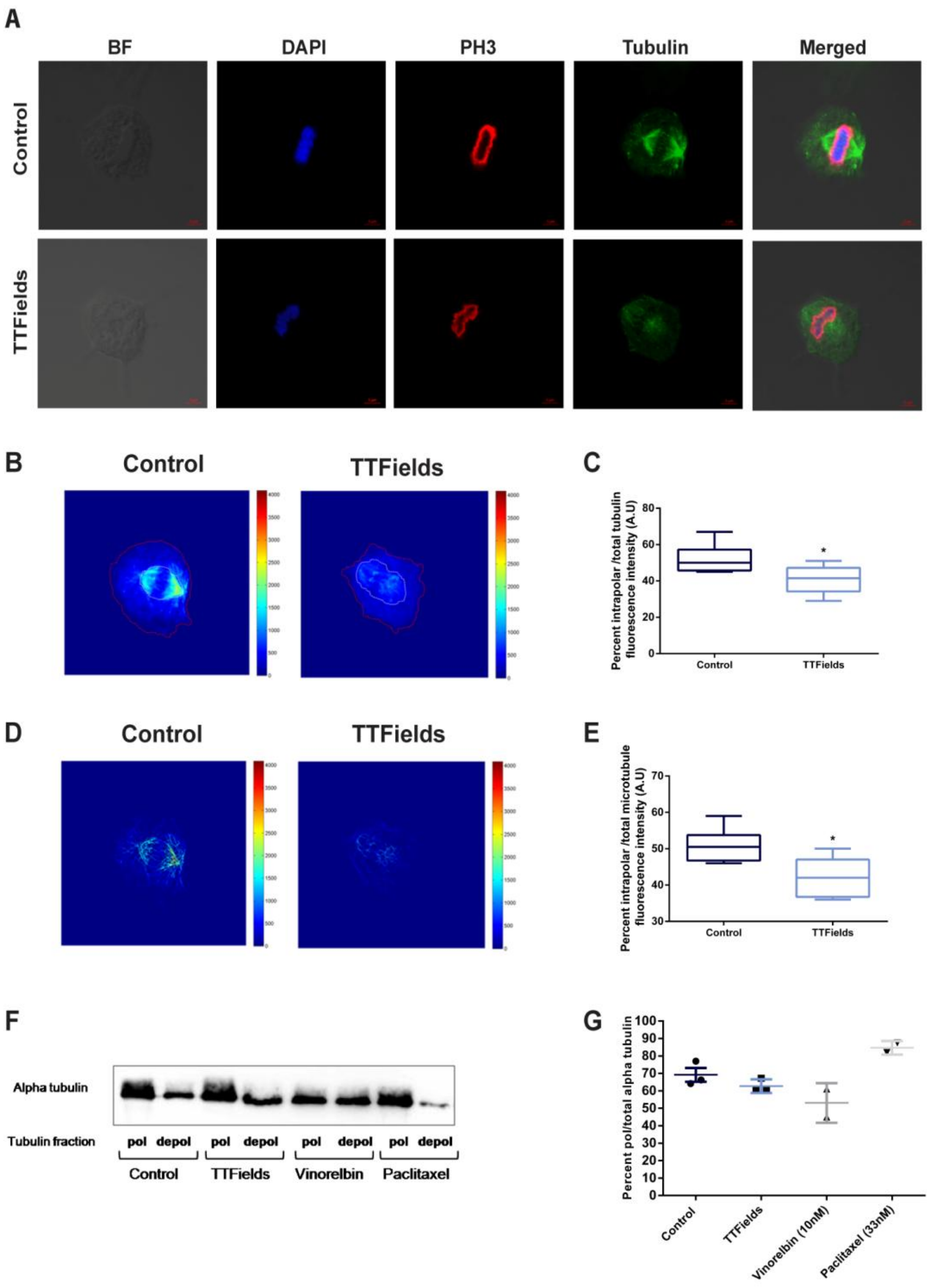

Supplementary Figure S1, related to Figure 1: (A-C) MDA-MB-231 cells were treated with TTFields for 24 h. (A) Confocal fluorescence microscopy images of: Upper panel- normal

metaphase alignment of chromosomes in control cell, Lower panel: metaphase alignment in TTFields treated cell. Blue, Dapi-stained DNA; Red, Phospho Histone 3(PH3stained chromosomes; green, tubulin. The scale bar represents 5 µm. (B) Tubulin fluorescence images were inverted and pseudocolored so that increasing fluorescence intensity is indicated from blue to red (scale bar represent arbitrary units). Dashed lines signify the region defined by the spindle and poles (white) and overall tubulin fluorescence within the cell (Red). (C) Box-and-whiskers plots show the percentage of fluorescence intensity defined by the spindle and poles in relation to the total sum of tubulin fluorescence within the cell. The boxes show the mean and the interquartile ranges, while the whiskers show the full range. (D) Z-stack based 2D visualization of the mitotic spindle structure in each cell following enhancement of the cytoskeletal filament networks. (E) Box-and-whiskers plots show the percentage of fluorescence intensity defined by the spindle and poles in relation to the total sum of microtubule fluorescence within the cell. (F-G) In a separate experiment cell lysets of U-87MG were collected following 24 h treatment with TTFields. (F) Immunoblot of polymerized (pol) and depolymerized (depol) tubulin fractions were compared to untreated cells and cells treated with Paclitaxel or Vinorelbine. (G) Graph represents the percentages of polymerized microtubules in relation to total tubulin. Horizontal bars indicate mean values with SD. 0.05 > *p > 0.01 from control group.

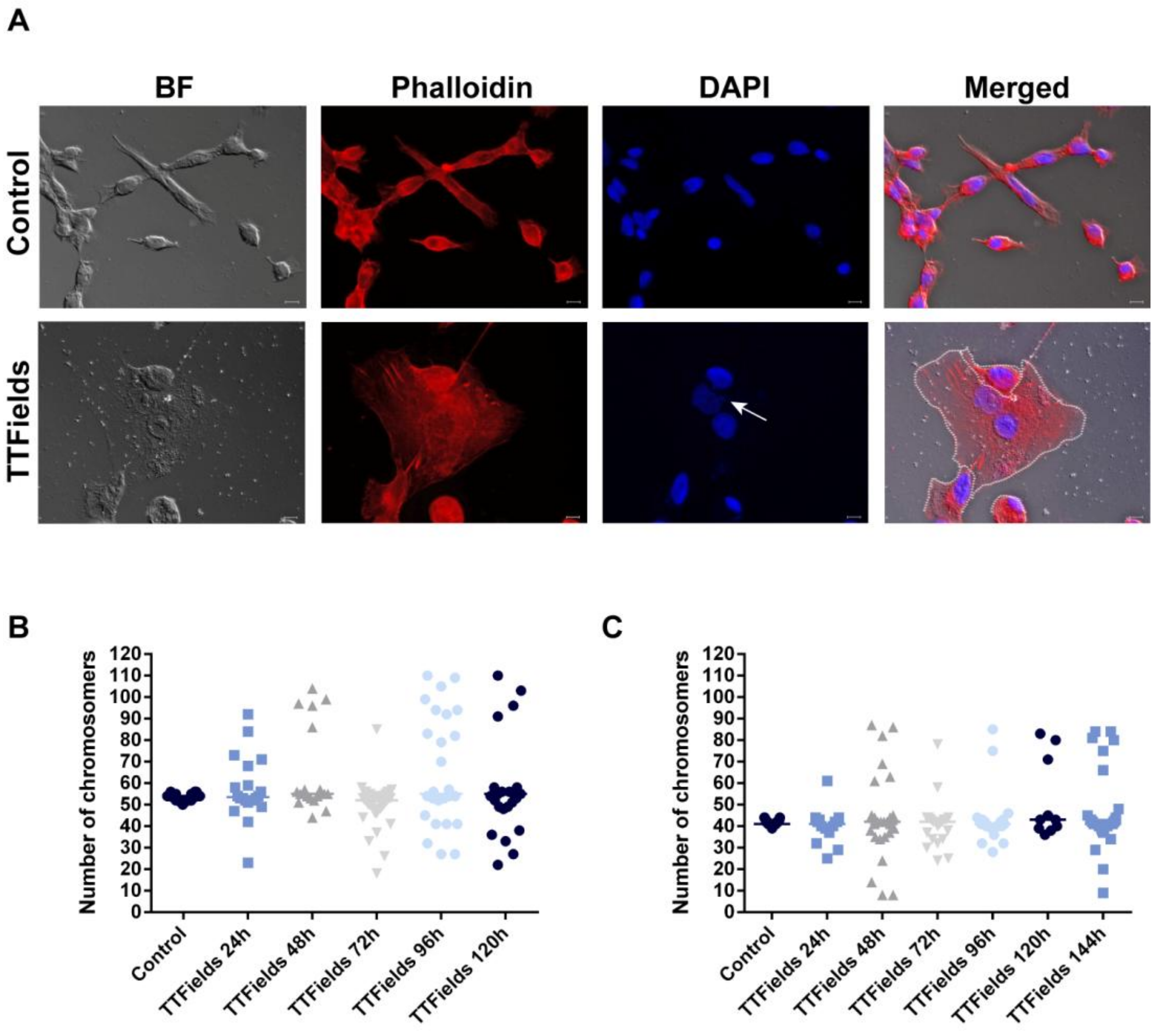

Supplementary Figure S2, related to Figure 2: MSTO-211H cells were treated with TTFields for 72 hours. (A) Upper panel, MSTO-211H control cell. Lower panel, TTFields treated cell. Arrow indicates micronuclei structures. Blue, Dapi-stained DNA; Red, Phalloidin-stained Actin. The scale bar represents 10 µm. In a separate experiment U-87 MG (B) and AsPC-1 (C) cells were treated with TTFields for 144h and 120h, respectively. Chromosome number was evaluated every 24 hr. Horizontal bars indicate median values (U-87 MG, p=0.0038; AsPC-1, p=0.0005; Brown-Forsythe test).

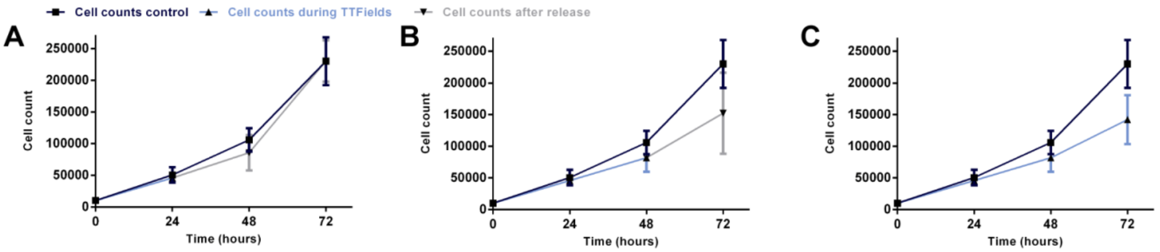

Supplementary Figure S3, related to Figure 4: A2780 cells treated with TTFields were released from treatment following (A) 24 h (p=0.2924 at 72hr), (B) 48 h (p=0.00185 at 72hr), and (C) 72 h (p=0.020 at 72hr). Cell counts were monitored 24 h and 48 h after release.

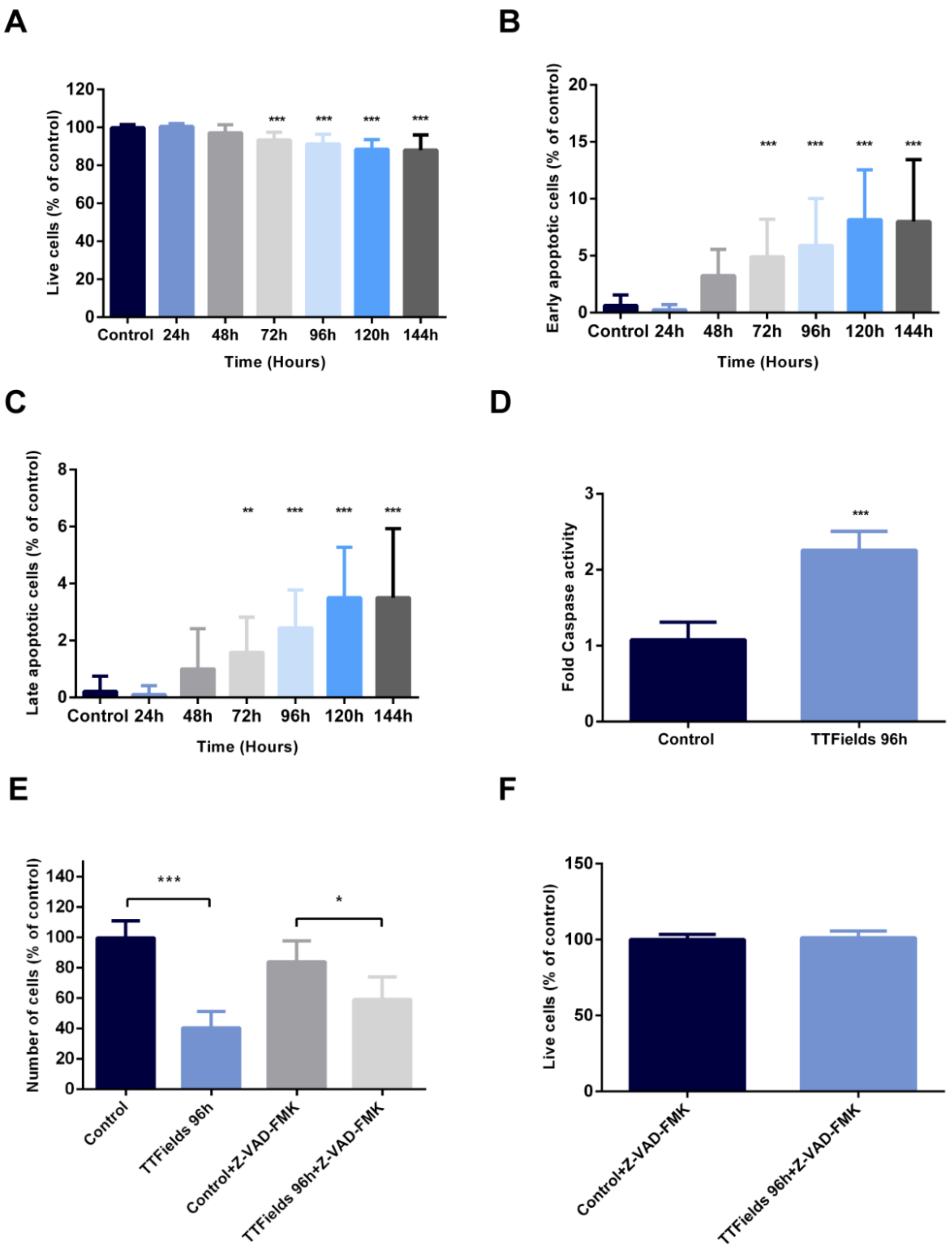

Supplementary Figure S4, related to Figure 6: U-87 MG cells were treated with TTFields for 144 hours. (A-C) Cell samples were collected and evaluated for apoptosis. (A) Live cells. (B) Early apoptotic cells. (C) Late apoptotic cells. (D) Caspase activity was evaluated using flow cytometry analysis following 96 hours of TTFields application. (E-F) Effect of pan-caspase

inhibition by Z-VAD-FMK on U-87 MG response to TTFields treatment. (E) Evaluation of apoptosis (F) Cell count. **p < 0.01, and ***p < 0.001 from control group.

Supplementary Table S1. Values of the electric properties of media used in this study

	DMEM	RPMI	EMEM
**pH***	7.0-7.4	7.0-7.6	7.0-7.4
**Ionic strength (mol/m^3)**	313	328	315
**Resistivity (Ω·m)**	0.70	0.74	0.71
**Current density (A/m^2)**	249.95	235.58	245.04

***According to ATCC**

Supplementary Movie S1. A cell expressing tubulin-GFP and treated with TTFields successfully completes cytokinesis which results in the formation of single viable binucleated progeny.

Supplementary Movie S2. A cell expressing tubulin-GFP and treated with TTFields successfully completes cytokinesis which results in apoptosis of both progeny.
